# Double migration of the endangered *Tricyrtis formosana* (Liliaceae) in Japan

**DOI:** 10.1038/s41598-024-51431-x

**Published:** 2024-01-10

**Authors:** Kaori Tsunenari, Takuro Ito, Masatsugu Yokota, Mayu Shibabayashi, Chiharu Endo, Kuo-Fang Chung, Yoshihisa Suyama, Ayumi Matsuo, Atsushi Abe, Akiyo Naiki, Hiroaki Setoguchi, Takashi Makino, Yuji Isagi

**Affiliations:** 1 Graduate School of Agriculture, Kyoto University, Kyoto, Japan; 2 Japan Broadcasting Cooporation, Tokyo, Japan; 3 The Center for Academic Resources and Archives, Tohoku University, Sendai, Japan; 4 Faculty of Science, University of the Ryukyus, Nishihara, Japan; 5 School of Forestry and Resource Conservation, National Taiwan University, Taipei, Taiwan; 6 Biodiversity Research Center, Academia Sinica, Taipei, Taiwan; 7 Graduate School of Agricultural Science, Tohoku University, Sendai, Japan; 8 Okinawa Churashima Foundation Research Institute, Botanical Laboratory, Okinawa, Japan; 9 Tropical Biosphere Research Center, University of the Ryukyus, Taketomi, Okinawa, Japan; 10 Graduate School of Human and Environmental Studies, Kyoto University, Kyoto, Japan; 11 Graduate School of Life Science, Tohoku University, Sendai, Japan

**Keywords:** Ecology, Biodiversity, Biogeography, Conservation biology

## Abstract

The Ryukyu Islands of Japan are a biodiversity hotspot due to geographical and historical factors. *Tricyrtis formosana* is a perennial herbaceous plant that commonly found in Taiwan. But only a few populations have been identified in a limited habitat on Iriomote Island, while populations of unknown origin occur near human settlements in an area on the main island of Okinawa. To better understand these populations of the phylogenetic uniqueness and intrinsic vulnerability, we conducted comparative analyses including (1) phylogeny and population structure with MIG-seq data, (2) photosynthesis-related traits of plants grown under common conditions and (3) transcriptome analysis to detect deleterious variations. Results revealed that *T. formosana* was split into two clades by the congeners and that Iriomote and Okinawa populations independently derived from ancestral Taiwanese populations in each clade. Photosynthetic efficiency was lowest in the Iriomote population, followed by Okinawa and Taiwan. Transcriptome analysis showed that the Iriomote population accumulated more deleterious variations, suggesting intrinsic vulnerability. These results indicate that each *T. formosana* population in Japan is phylogenetically unique and has been independently dispersed from Taiwan, and that the Iriomote population presents a high conservation difficulty with a unique photosynthesis-related characteristic and a larger amount of deleterious variations.

## Introduction

The Ryukyu Archipelago and Taiwan, located along the western rim of the Pacific Ocean, comprise of approximately 140 subtropical islands and are home to many endemic species, including those listed as CR (Critically Endangered), EN (Endangered), or VU (Vulnerable) on the ICUN Red List, as well as many rare plants. The diverse floristic composition of the Ryukyu Archipelago can be attributed to multiple factors. This region overlaps with the migration routes of birds between the northern and southern hemispheres and is impacted by the Kuroshio Current, a warm current known to facilitate overseas dispersal of plant species^1,2^. Additionally, the Ryukyu Archipelago is regularly exposed to strong tropical storms originating from the east of the Philippines. The diverse flora of the Ryukyu Archipelago is the result of multiple factors, including the introduction of various plant taxa through different migration routes and vectors. The complexity of its geological history also plays a role in its diversity. The island arc was separated from the eastern edge of the continent during the late Miocene and early Pleistocene and underwent repeated separations and connections with neighboring islands due to marine regression-transgression associated with the glacial and interglacial cycles of the Pleistocene^3–7^. The geological history played a key role in shaping its diverse flora of the Ryukyu Archipelago, and made the area a habitat of species that have survived under isolated conditions^8^ as well as new endemic species that have emerged as a result of adaptive differentiation^9^.

Iriomote Island, locating in the southern part of the Ryukyu Archipelago, houses 1,165 species of vascular plants over an area of 289.61 km^2^, with 59 species (5%) being endemic^10^. Despite the lower proportion of plant endemicity compared to Hawaii archipelago (90%) and Galapagos Archipelago (approx. 35%)^11^. Iriomote shows a larger number of endemic species per area (4.02 km^−2^) compared to Hawaii (0.06 km^−2^) and Galapagos Archipelago (0.022 km^−2^)^10,11^. This highlights the high biodiversity of Iriomote and the variety of species that thrive on the small island.

This study focused on a perennial herbaceous plant, *Tricyrtis formosana* Baker (Lilliaceae), as a representative example of plant differentiation influenced by the complex environmental and geological conditions of the Ryukyu Archipelago. The genus *Tricyrtis* commonly known as ‘Toad lily’ and is endemic to eastern Asia, including the Himalayas, China, Japan, Taiwan, and the Philippines^12^. There are four species of *Tricyrtis* in Taiwan, of which only *T. formosana* is found on Iriomote Island and Okinawa Island of the Ryukyu Archipelago in Japan^13^. In Taiwan, *T. formosana* is a widespread and commonly found plant^13,14^, and is widely cultivated for horticultural purposes in Japan. In contrast, only a few wild populations are known in Iriomote and Okinawa Island.

Based on the distinct habitat and petal colour, *T. formosana* individuals in the Iriomote population can be distinguished from *T. formosana* that grows in Taiwan. The Iriomote population is known to grow in limited habitats, primarily near waterfalls under constant spray conditions, with only a small number of individuals estimated to be about one hundred found in five locations. Due to its restricted distribution, this species is listed on the Red List of the Japanese Ministry of the Environment^15^.

Meanwhile, the population on Okinawa Island, the central Ryukyu Archipelago, is distributed near human settlements, and its population size is much larger than that of Iriomote. Okinawa Island, located 400 km northeast of Iriomote Island, represents the northern marginal distribution of this species. Although being more distant from the Taiwan mainland than Iriomote Island, the growing environment and petal colour of the Okinawa population are similar to those of the Taiwanese population. This raises the possibility that the Okinawa population was introduced from the Taiwanese population by human activities, and the conservation value of this population has not yet been determined.

**Figure 1:**
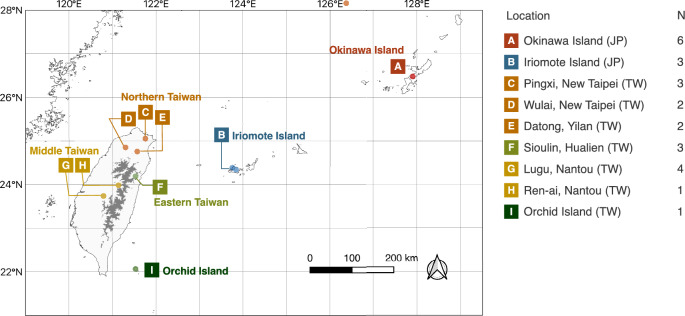
Locations of sampling sites of *T. formosana* with a legend indicating the number of samples per locations for MIG-seq. The gray shading in Taiwan represents areas with elevations of 2000 m or higher, which may act as barriers to the distribution of *T. formosana*. Shorelines were extracted from GSHHG^17^, and the contour lines of 2000 m were generated from Digital Elevation Model of ETOPO 2022^18^. These vector data were integrated, and the map was created using QGIS 3.22^19^.

The objective of this study was to investigate the intraspecific phylogeny and physiological traits of populations *T. formosana*. The focus was on understanding the origin and assessing their conservation value based on the uniqueness of the rare populations known in the isolated and marginal distribution range of the species in Japan (Fig. 1). *T. formosana* comprises populations with varying physiological characteristics: the commonly found Taiwanese populations growing in forests, the Okinawa population growing along farm roads and in forests near human settlements, and the Iriomote population growing only near waterfalls in forests exposed to constant spray. These differences in growth conditions suggest that each population has different physiological and genomic characteristics. Some endangered species exhibit intrinsic vulnerability due to genomic factors, such as a high accumulation of deleterious mutations and a smaller ratio of duplicated genes, compared with non-endangered congener species, which increase their conservation difficulty^16^. To guide the appropriate direction of conservation strategies, we integrated different sources of information of phylogeny, physiological characteristics and genomic traits.

## Results

### Phylogenetic analysis and population structure analysis

**Figure 2:**
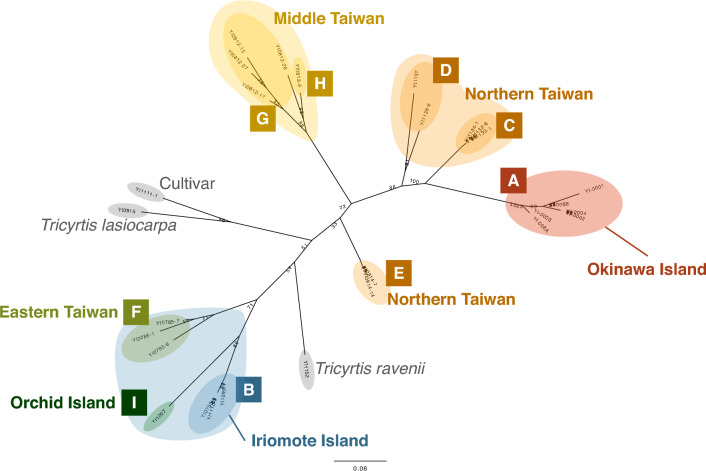
ML phylogenetic tree of *T. formosana* based on 233 SNP loci obtained by MIG-seq analysis using TVMef model. Labels (**A**–**I**) correspond to those in Fig. 1.

A taxon that has been considered *T. formosana* s. l. was clearly divided into two clades by the intervention of congener species: *T. ravenii* and *T. lasiocarpa* (Fig. 2). The first clade consists of samples from populations in the eastern Taiwan, Orchid Island, and Iriomote Island and the other clade consists of those in Okinawa Island, the northern Taiwan and the middle Taiwan populations. The populations in Iriomote and Okinawa, Japan were found to be distantly related, with the Okinawa population being closer to northern Taiwan and distinct from both the Iriomote population and a cultivar commonly sold as *T. formosana* in Japan. For the clade consisting of A, C, D, G, H, and E, *T. lasiocarpa* was an outgroup, and population A on Okinawa Island was in the most derived position. For the clade consisting of B, F, and I, T. ravenii was an outgroup, and Iriomote population B was in a more derived position than Taiwanese population E.

The long branch separating Okinawa from northern Taiwan and long inner branching of the Okinawa population suggest it has a natural, long-standing presence on the main island of Okinawa. Conversely, the Iriomote population is closely related to the Orchid Island population and the inner branches are short (Fig. 2).

The STRUCTURE HARVESTER showed that the delta K was highest at K = 2, followed by K = 3, 7, and 4 (Supplementary Fig. S1). The results of the STRUCTURE analysis indicated that at K = 2, the Okinawa population and the Iriomote population belonged to distinct clusters. As the number of clusters increased, the population genetic structure became more intricate, but these patterns corresponded to the internal structure of the phylogenetic tree shown in Fig. 2. The results of the STRUCTURE and phylogenetic analyses were consistent and did not contradict each other. At K = 3, the *F*_ST_ values were as follows: Cluster 1 = 0.5319, Cluster 2 = 0.2824, and Cluster 3 = 0.6326. The fact that Cluster 1 and Cluster 3 including Iriomote and Okinawa populations, respectively, have values greater than that of Cluster 2, which corresponds to Middle Taiwan, indicates that the two Japanese populations are both distinct and derived from Taiwan.

The observed heterozygosity at all base positions calculated with populations in Stacks 2.55 was lower in the Japanese populations; 1.0 × 10^–4^ in the Iriomote population, 1.4 × 10^–4^ in the Okinawa population and 3.2 × 10^–4^ in the Taiwanese population.

### Comparative analysis of photosynthesis-related traits

**Figure 3:**
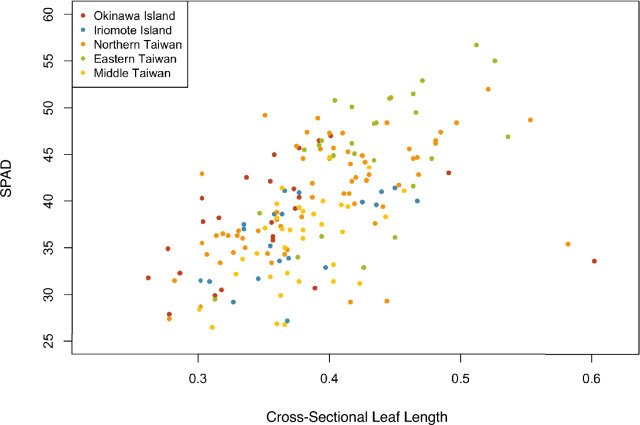
SPAD (chlorophyll content) and cross-sectional leaf length.

The results of the linear mixed model indicated a positive correlation between SPAD and cross-sectional length (LMM, coefficient = 56.65, *t* = 4.76). Additionally, the results of the GLM multiple regression analysis revealed a significant positive effect for cross-sectional length (coefficient = 61.6610, *t* = 9.114, *p* < 0.001) while the effect for the population was not significant (Fig. 3).

**Figure 4:**
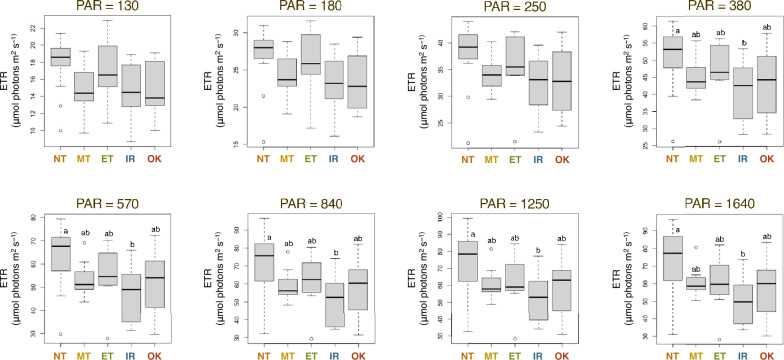
ETR (electron transportation rate) calculated at each PAR (photosynthetically active radiation) = 130–1640 mol quanta m^−2^ s^−1^. *NT* Northern Taiwan, *MT* Middle Taiwan, *ET* Eastern Taiwan, *IR* Iriomote, *OK* Okinawa population.

Multiple comparisons revealed a significant difference in ETR (electron transportation rate in photosystem II) between the Northern Taiwan and the Iriomote populations at PAR ≥ 380 mol quanta m^−2^ s^−1^ (*p* < 0.05) (Fig. 4). Despite the absence of a significant difference, the overall ETR was the highest in the Taiwanese population, followed by the Okinawa and Iriomote populations.

### Estimation of conservation difficulty by transcriptome analysis

**Figure 5:**
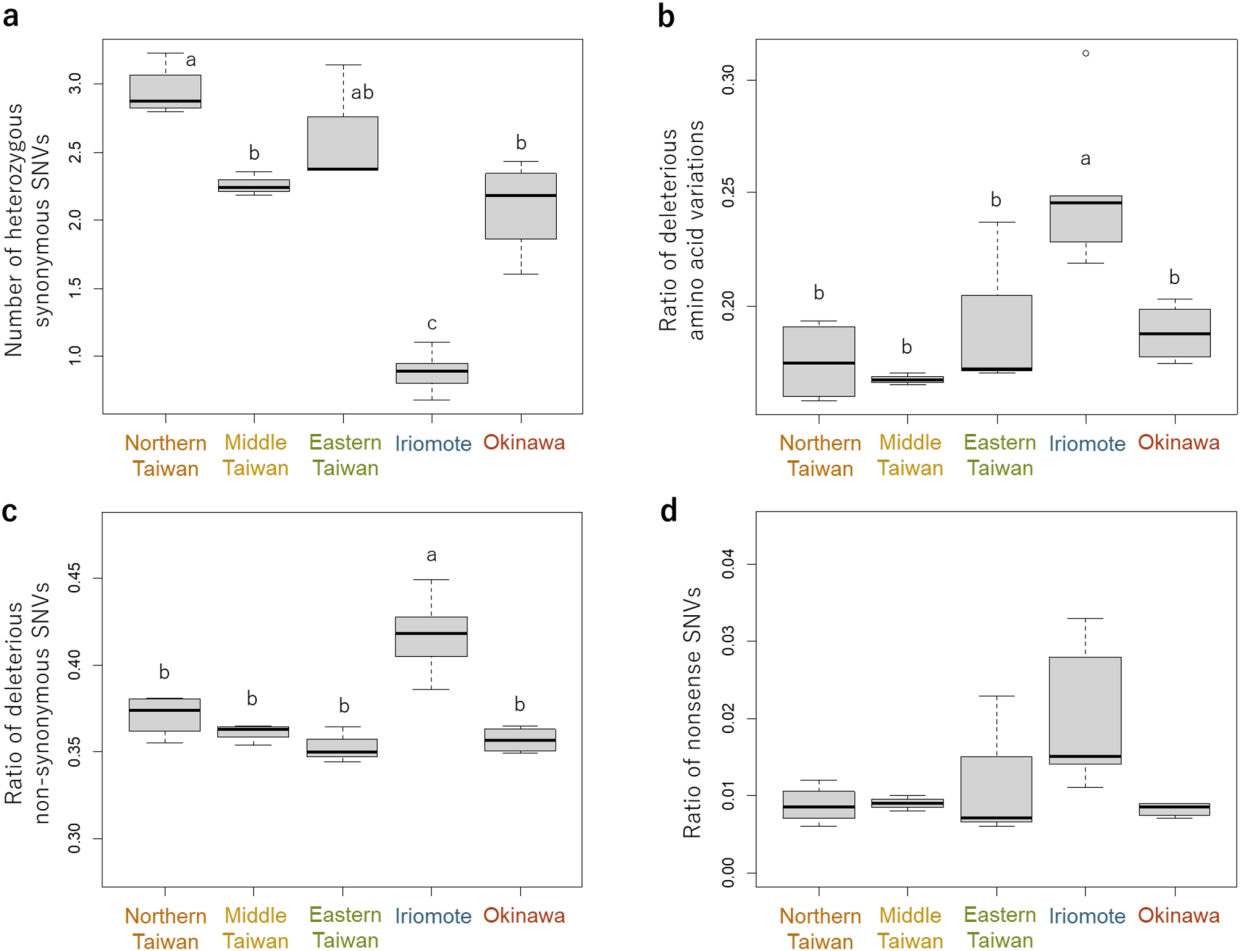
Box plots of genetic characteristics of the Taiwanese (northern, middle and eastern), Iriomote and Okinawa populations based on transcriptome analysis. Different alphabets indicate significant differences. (**A**) Genetic diversity based on the number of heterozygous synonymous SNVs per kb on the longest coding sequences, (**B**) ratios of deleterious amino acid variations in heterozygous SNVs estimated using PROVEAN, (**C**) ratios of non-synonymous SNVs calculated for each gene, (**D**) ratio of nonsense SNVs in the total non-synonymous SNVs.

The evaluation of genetic diversity based on the number of heterozygous synonymous SNVs in the transcripts per kb revealed that individuals in the Iriomote population exhibited significantly lower diversity compared to other populations (p < 0.05) (Fig. 5A). Individuals in the Iriomote population exhibited significantly higher values in three indices of deleterious variations; the ratio of deleterious amino acid variations in heterozygous SNVs (Fig. 5B), the proportion of non-synonymous variants for each coding sequence (Fig. 5C), and the proportion of nonsense SNVs to total non-synonymous SNVs (Fig. 5D), compared to the Taiwan and Okinawa population. Individuals of the Taiwan and Okinawa populations exhibited lower rates of detrimental mutations, which corresponds to the fact that these individuals are robust and adapted to a wider range of environments in the wild.

## Discussion

As for the taxonomic treatment of *Trycyrtis* in Taiwan, The 1st edition of the Flora of Taiwan recognizes a total of 5 species and 2 varieties of *Trycyrtis* plants in Taiwan^20^, which include *T. formosana* var. *formosana*, *T. formosana* var. *glandosa*, *T. stolonifera*, *T. ovatifolia*, *T. lasiocarpa*, and *T. suzukii*, while in the 2nd edition of the Flora of Taiwan, two species and five varieties of *Tricyrtis* are recognized^21^, including *T. formosana* var. *formosana*, *T. formosana* var. *glandiflorum*, *T. formosana* var. *stolonifera*, *T. formosana* var. *ovatifolia*, *T. formosana* var. *lasiocarpa*, and *T. suzukii.* Subsequently, a taxonomic reevaluation of the varieties of *T. formosana* had classified evergreen plants with traits of glabrous ovaries/capsules, primarily found in low-elevation areas, as *T. formosana*, and also introduced a new species, *T. ravenii,* for plants primarily distributed in high-elevation areas exhibiting deciduous characteristics and hairy ovaries/capsules^13^. Furthermore, due to distinct differences in leaf morphology, flower and fruit characteristics, *T. formosana* var. *lasiocarpa* was recognized as an independent species, *T. lasiocarpa*. Following the revisions, the *Tricyrtis* genus in Taiwan is currently recognized to consist of four species: *T. formosana*, *T. ravenii*, *T. lasiocarpa*, and *T. suzukii*^13,22^.

In this study, ML phylogenetic tree revealed that *T. formosana* was divided into two distinct clades by the intervention of two congener species, and each of them may potentially represent separate species (Fig. 2). However, we have not identified any distinguishing morphological characteristics to differentiate between these two clades. The taxonomic treatment of these two clades, and integrated understanding of phylogenetic and morphological differentiation are matters that should be addressed in the future. The phylogenic clusters of the Taiwanese population of *T. formosana* corresponded to the geographic distribution; however, the two populations in Japan did not have the closest phylogenetical relationship to each other; deriving from different clades in Taiwan. Both the Iriomote and Okinawa populations had a long branching from the most related Taiwanese population, indicating their phylogenetic uniqueness.

**Figure 6:**
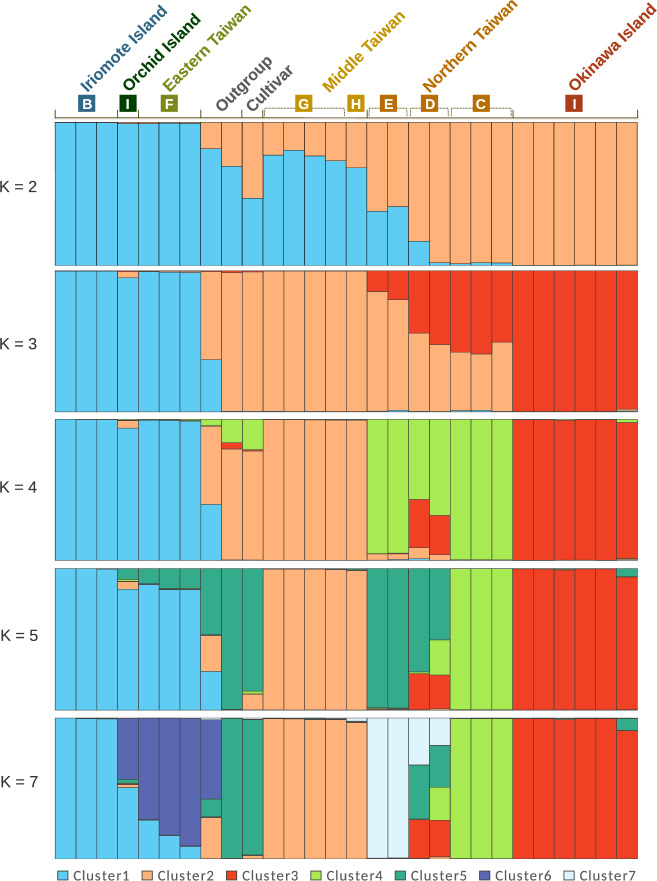
Population structure of the 28 populations of *T. formosana* estimated by STRUCTURE analysis. The mean Fst at K = 3; Cluster 1 = 0.5319, Cluster 2 = 0.2824, Cluster 3 = 0.6326.

In accordance with the phylogenetic tree, the STRUCTURE analysis split the ancestral genetic cluster into a consistent pattern (Fig. 6), suggesting that the Okinawa population was derived from the northern Taiwan population, and the Iriomote population has the closet phylogenetic relationship with the eastern Taiwan and Orchid Island population. Based on the mean *F*st values at *K* = 3 in STRUCUTRE, the ancestor of *T. formosana* (*F*st = 0.2824 for Cluster 2 in the panel of *K* = 3, Fig. 6) is likely to have originated from a population that has diverged from congener species in the middle of the Taiwan mainland. Then the population have expanded the distribution to the northern (*F*st = 0.6326 for Cluster 3 in the panel of *K* = 3, Fig. 6) and the eastern (*F*st = 0.5319 for Cluster 1 in the panel of *K* = 3, Fig. 6) areas, followed by dispersal to Okinawa Island from the northern area, as well as Iriomote Island from the eastern area. It is noted that *T. formosana* is distributed from 100 to 1500 m sea level in Taiwan mainland^15^, and the populations of Northern Taiwan, Eastern Taiwan and Middle Taiwan in this study are separated from each other by 2000–3000 m mountainous ranges that can act as geographical barrier.

The two Japanese populations were phylogenetically distinct (Fig. 2), and it is likely to have arrived in Japan independently through separate and distinct routes; from the northern area in Taiwan to Okinawa Island, and from the eastern area in Taiwan to Iriomote Island via Orchid Island. Thus, the Japanese populations of *T. formosana* are distinctive from each other in their phylogenetic position as well as in their migratory pathways, and each has separate conservation values.

The population of *T. formosana* in Okinawa presents a case of disjunct distribution, as it is inferred to have originated from northern Taiwan, located approximately 600 km away. While the region experiences high-intensity tropical storms, which could provide strong winds capable of seed dispersal, the seeds lack the aerodynamic structure necessary for successful dispersal through wind. Dispersal by sea currents via rafting is considered unlikely due to the lack of floatability and saltwater tolerance of the seed, which are necessary for long-distance dispersal by sea currents. The small and flat seeds of *T. formosana*, measuring less than 2 mm square, make it an ideal candidate for dispersal by birds. Phylogenetic studies of *Solenogyne mikadoi* (Asteraceae) and *Lobelia loochooensis* (Campanulaceae) support the hypothesis of avian-mediated dispersal, as the antitropical distribution of these species suggests the presence of migratory birds in more than 7000 km distance from Australia to the Ryukyu Archipelago^23,24^.

Several instances are recognized where different closely related taxa are distributed and have undergone differentiation across various regions of the Ryukyu Islands. *Tashiroea okinawensis*/*T. yaeyamensis* (Melastomataceae) exhibits shallow differentiation in the Ryukyu region, with closely related species found on the Chinese mainland^25^. *Viola iwagawae*/*V. tashiroi* (Violaceae) show minor differentiation in this area^26^. The globally widespread species *Dodonaea viscosa* (Sapindaceae) has lineages near Taiwan on Ishigaki Island in the southern Ryukyus while lineages closer to the Philippines on the islands in the northern and central Ryukyus^27^. While molecular phylogenetic analysis has not been conducted, taxa such as *Eurya zigzag*/*E. yaeyamensis* (Pentaphylacaceae), *Adinandra ryukyuensis*/*A. yaeyamensis* (Pentaphylaceae), and *Swertia tashoroi*/*S. makinoana* (Gentianaceae) exhibit similar distributions in this area. Comparative analyses of these taxa could provide valuable information on the formation processes of biodiversity in this region.

The discovery that the Orchid Island population is the most closely related to the Iriomote Island population, rather than the geographically proximate northern Taiwanese population, is an interesting finding. Moreover, it is intriguing to note that *Freycinetia williamsii* (Pandanaceae), *Nothapodytes foetida* (Icacinaceae), *Monoon liukiuensis* (Annonaceae), which are critically endangered plant species restricted to Iriomote Island in Japan, are not distributed on the main island of Taiwan but on Orchid Island. This observation raises the possibility of an unknown biological factor that could be linking Iriomote Island and Orchid Island. Further investigation is necessary to elucidate the underlying mechanisms responsible for this pattern, and such research could provide valuable insights into the distribution of rare plant species in the region.

Despite the Okinawa population being sampled within a smaller range of 150 m stratch along the rural road, compared to the Iriomote populations, which were separated more than 10 km, we found that the genetic diversity assessed by number of heterozygous synonymous SNVs per kb was about twice as high in the Okinawa population (Fig. 5A). The greater genetic diversity of the Okinawa population may be due to its larger population size compared to that of the Iriomote population. The high genetic diversity of the Okinawa population is crucial for its survival, as it may provide a foundation for the species to adapt to future environmental changes^28^.

The conservation value was recognized on both population of the *T. formosana* in Iriomote Island and Okinawa Island for their own uniqueness. However, we indicate that the Iriomote population was characterized higher vulnerability as evidenced by its the lower genetic diversity (Fig. 5A) and larger accumulation of deleterious variants (Fig. 5B–D) compared to other populations. The genome-wide reduction of genetic diversity and accumulation of deleterious variants in functional genes might result in the narrow habitat range and high vulnerability for the Iriomote population, which is limited only to the vicinity of a waterfall. Previous studies have shown that population isolation and contraction can weaken purifying selection and increase the number of deleterious mutations in wild species, such as the brown eared pheasant (*Crossoptilon mantchuricum*) and two subspecies of mountain gorilla (*Gorilla* spp.)^29,30^. This trend is also expected to be observed in the Iriomote population due to its narrow habitat range and small population size. However, inbreeding can sometimes lead to purging of deleterious recessive mutations in a small wild population. While it has been observed that inbreeding reduces the burden of deleterious mutation^31,32^, it can not necessarily apply to extremely small populations, because alleles may fall out of purifying selection, leading to population decline and the accumulation of deleterious mutations^33,34^. In contrast, the Okinawa population shows less loss of genetic diversity and accumulation of deleterious mutations, likely due to its larger population size. This is supported by information in the Okinawa prefecture Red List^35^, which states that *T. formosana* in Okinawa Island is widely distributed throughout the upper of mountain range in the same water catchment area.

Previous research presented a relationship between photosynthesis efficiency, as indicated by PAR (photosynthetically active radiation) absorption, and levels of net primary production^36^. The ETR (electron transportation rate) is a measure of light energy and the decrease leads to photoinhibition, which is characterized by damage to the reaction center chlorophyll^37^ and a decline in photosynthetic efficiency. Our study found that the ETR values of the Iriomote population were significantly lower than those of the Northern Taiwan population when PAR was greater than 380 (Fig. 4). Shade plants typically have lower ETR compared to sun plants, which is consistent with the fact that the Iriomote population is restricted to the vicinity of the waterfall in the forest. The Iriomote population is considered a critically endangered species on the Red List, Ministry of Environment of Japan^2^. To ensure the conservation of this population, care must be taken to prevent photoinhibition under intense sunlight, as shade plants are susceptible to an excess of light. On the contrary, the Okinawa population did not show a reduction in genetic diversity or an accumulation of deleterious variants, hence it is considered that the population can be conserved by maintaining the status quo, that is, relatively simple management, such as the removal of competing herbaceous species in the habitat.

In conclusion, our study has shown that the two Japanese populations of *T. formosana* are phylogenetically distinct and have high conservation values due to their independent migration from Taiwan. In Japan, the Act on Conservation of Endangered Species of Wild Fauna and Flora calls for maximum conservation efforts for species that is designated as nationally rare and endangered. While *T. formosana* in Japan has not yet been designated as such, our results suggest that the Iriomote population deserves this recognition. Furthermore, our findings indicate that the Iriomote population is more difficult to conserve compared to other populations, due to its photophysiological traits and accumulation of deleterious mutations in the genome. These results provide a basis for future conservation and breeding programs and highlight the need for more careful management of plants from the Iriomote populations under ex-situ conditions, including protection measures to prevent photoinhibition. Our study provides insight into the phylogenetic origin and uniqueness of endangered *T. formosana* in Japan and evaluates the conservation difficulty of each population. The distribution of plant species and administrative divisions, such as national borders, are independent. Many plant species have a broad geographic range that spans multiple countries and, as a result, may be deemed rare in a particular country or region. By correctly evaluating the phylogenetic distinctiveness and genomic features of plant populations in each region through methods like those used in this study, we can assess the conservation status of these species and develop effective conservation strategies.

## Materials and methods

### Sample collection and DNA extraction

Leaf samples were collected from Okinawa Island, Iriomote Island, Taiwan mainland and Orchid Island. One marketed cultivar and two outgroups (one each of *T. lasiocarpa* and *T. ravenii*) were also used for the analysis (Supplementary Table S1). The collection and use of plant materials in this study were carried out in compliance with relevant institutional, national, and international guidelines and legislation. Necessary permissions and licenses were obtained from the relevant authorities (Ministry of the Environment and Forestry Agency for Japanese samples, and Academia Sinica for Taiwanese samples). The plant specimens were identified by two of the current authors, Akiyo Naiki, an associate professor at University of the Ryukyus and Takuro Ito, an assistant professor at Tohoku University and voucher specimens were deposited at the Herbarium of the Faculty of Science, University of the Ryukyus (RYU; Naiki 18254 and Naiki 18246) and the Herbarium of Tohoku University (TUS; T. Ito 4674, T. Ito 4915, T. Ito 4928, T. Ito 5019, T. Ito 5021, T. Ito 6126 and T. Ito 8800). Genomic DNA was extracted from a total of 28 leaf samples using a modified CTAB method^38^.

### SNP genotyping by MIG-seq

DNA samples were sequenced by MIG-seq method following the protocol of Suyama and Matsuki^39^. MIG-seq library were constructed through two PCR steps. First, multiple nonrepetitive regions from various ISSRs were amplified by multiplexing with tailed ISSR primers. PCR was performed in an AB Veriti™ 96-Well Thermal Cycler (Thermo Fisher Scientific, Applied Biosystem, CA, USA) under the following thermocycler conditions: initial denaturation at 94 °C for 1 min; followed by 30 cycles of denaturation at 94 °C for 30 s, annealing at 38 °C for 1 min and extension at 72 °C for 1 min. A final extension at 72 °C for 10 min completed the first PCR. Second, we cleaned and normalized each 1st PCR product using Short read eliminator (SRE) kit (Pacbio, London, UK), followed by a second PCR according to the original protocol. We did purification and size selection (400–800 bp) with SPRIselect (Beckman Coulter, Brea, CA, USA). We confirmed successful library preparation using the Microchip Electrophoresis System for DNA/RNA Analysis MCE®-202 MultiNA with the DNA-2500 Reagent Kit (Shimadzu, Kyoto, Japan) following the manufacturer’s protocol. Approximately 12 pM of each library was sequenced on an Illumina MiSeq platform (Illumina, San Diego, CA, USA).

Raw reads were trimmed, and quality filtered (CROP: 150, HEADCROP: 20, SLIDING WINDOW: 4: 15, MINLEN: 130) with trimmomatic-0.38 software^40^.

De novo SNP calling was conducted with denovo_map.pl pipeline in Stacks 2.55 software^41^. The optimization parameters were set as follows: minimum depth of coverage = 3, number of mismatches allowed between stacks within individuals = 2. Population structure analysis was performed using the populations pipeline with following parameters; the number of populations = 1, the minimum percentage of individuals in a population = 0.1, minimum minor allele frequency = 0.05, and maximum observed heterozygosity = 0.5. We calculated observed heterozygosity as an output option of the populations pipeline. The SNP data was filtered using Tassel 5 software^42^, selecting SNPs shared by at least 60% of individuals and with a probability of 10% of being shared by these individuals. For each sample, we obtained 59,876 to 156,382 reads (average: 113,113), 3,194 to 6,693 loci (average: 5162) and 889 to 2031 (average: 2031) SNPs.

### Phylogenetic and population structure analyses

We employed ModelTest-NG software^43^ to determine the optimal nucleotide substitution model for constructing a maximum likelihood (ML) phylogenetic tree. Phylogenies were inferred using RAxML-NG 0.9.0 software^44^, with 100 bootstrap replicates to evaluate reliability of the topology. The result was visualized using FigTree 1.4.4 software (http://tree.bio.ed.ac.uk/software/figtree/).

We analyzed population structure of *T. formosana* using STRUCTURE v.2.3.4 software^45^. The population model was configured to allow admixture and the correlation of allele frequencies between clusters. Ten independent simulations were conducted for each K (K = 1–15) with 50,000 burn-in steps followed by 100,000 Markov Chain Monte Carlo steps. The Optimal value of K was estimated using STRUCTUR EHARVESTER^46^.

### Morphological and physiological traits

We evaluated chlorophyll content, cross-sectional length and electron transportation rate, as for traits related to photosynthetic capacity in this study. Chlorophyll content has been shown to be a reliable proxy for the maximum rate of carboxylation (Vcmax), which is a critical determinant of photosynthetic capacity^47^. Cross-sectional leaf length influences leaf robustness and is positively correlated with photosynthetic output and solar radiation per area^48^, making it a useful indicator of leaf structural durability and photosynthetic output^49^. ETR measures the consumption of light energy by electrons and a decrease in ETR results in an excess of light energy, causing photoinhibition and decreased photosynthetic efficiency^37^.

The geographical locations of *T. formosana* were classified into five categories: Okinawa Island (designated as A in Fig. 1), Iriomote Island (B), Northern Taiwan (C, D, E), Eastern Taiwan (F), and Middle Taiwan (G, H). These locations were used to evaluate photosynthesis-related traits, including chlorophyll content, cross-sectional length of leaves and electron transportation rate (ETR) in photosystem II. In addition chlorophyll content was indirectly inferred from SPAD values, which serve as an index of chlorophyll content^50,51^.

### Cross-sectional leaf length and chlorophyll content

Healthy, fully expanded leaves of individual plants from each location grown under common light intensity (ca. 300 mol quanta m^−2^ s^−1^), temperature (20 °C), daylength (10 h) and nutrition conditions in the laboratory for a minimum of two and half months were used for analysis (Table S2).

The SPAD Chlorophyll Meter (SPAD-502 Plus, CONICA MINOLTA) was used to measure the SPAD value on healthy portions of leaves. Three to five measurements were taken per leaf, and the average was used as the SPAD value for each leaf. The cross-sectional length of the leaves was determined by measuring the thickness of the same portion of the leaves using a thickness gauge.

The statistical analysis of the relationship between SPAD value and cross-sectional leaf length was performed using R v. 4.2.1. A linear mixed model regression analysis was carried out using lme4 package, with SPAD as the objective variable and cross-sectional length (fixed effect) and population (variable effect) as explanatory variables. Subsequently, a multiple regression analysis was performed using the GLM function with SPAD as the objective variable and population and cross-sectional length as explanatory variables.

### Electron transportation rate based on pulse amplitude modulated fluorometry method

Chlorophyll fluorescence analysis is a widely utilized technique for evaluating plant physiology as it provides a measure of the electron transport rate and overall photosynthesis through the proportion of light absorbed by chlorophyll^52^. We classified the same individuals at the same category as used in 5.2.2; Iriomote Island, Okinawa Island, Northern Taiwan, Eastern Taiwan and Middle Taiwan (Supplementary Table S2), and fully expanded healthy leaves from the upper part of the plants were selected for analysis. To make the electron accepter in reaction center chlorophyll of photosystem II an oxidized state, the leaves were pre-darkened by completely wrapping them in aluminum foil for at least 30 min. Chlorophyll fluorescence and electron transport rate were then measured using a PAM fluorometers (MINI-PAM-II, Walz).

Each sample was sequentially irradiated with photosynthetically effective radiation (PAR) of 0, 50, 90, 130, 180, 250, 380, 570, 840, 1250, 1640 mol quanta m^−2^ s^−1^, and the electron transfer rate (ETR)was calculated at each PAR. For the ETR values of the five categories, an analysis of variance was performed using ANOVA function of R for each PAR phase, and in the PAR phase where the results were significant, a multiple comparison test (correction for holm p-values) was then performed to determine which populations had significant differences between them.

### Estimation of conservation difficulty by transcriptome analysis

#### RNA extraction and de novo assembly

We extracted RNA of 19 samples from 2 Japanese and 3 Taiwanese populations (Supplementary Table S3) using an Agilent Plant RNA Isolation Mini kit (Agilent Technologies, Santa Clara, CA, USA), in accordance with the manufacturer’s protocols. Total RNA was sequenced on an Illumina NovaSeq 6,000 sequencer at 100 nucleotide paired-end (PE) reads. Low-quality reads, defined as those with more than 10% of the bases having a quality score below 30, were filtered using the FASTQ quality filter in the FASTX-Toolkit (http://hannonlab.cshl.edu/fastx_toolkit/). Five million PE reads were collected from each individual to minimize the influence of the difference in the number of reads among individuals, and they were used for de novo RNA-seq assembly by Trinity ver. 2.11.0^53^. In cases where multiple splicing isoforms for transcripts were obtained, the longest one with the same accession among the different isoforms in the Trinity assembly output was adopted.

#### Identification of heterozygous SNVs and calculation of genetic diversity

To identify heterozygous single nucleotide variants (SNVs) in each individual, the five million PE reads for each individual were mapped to the reference constructed by Trinity, using BWA version 0.7.13^54^. SNVs were then identified using SAMtools version 1.3^55^. The number of heterozygous synonymous SNVs with a minimum read depth of 10, which were deemed to be putative neutral variations indicative of genetic diversity, was calculated per kb of the longest transcript. The obtained values were compared among populations (i.e., Okinawa, Iriomote and Taiwan) using Kruskal–Wallis test (one-way ANOVA), and multiple comparisons were conducted as a post-hoc test, under Holm’s correction.

#### Estimation of deleterious amino acid variation

Using Protein Variant Effect Analyzer (PROVEAN^56^), we examined the non-synonymous SNVs that were homologous to plant amino acid sequences to judge whether each amino acid variation might affect protein function. By default, threshold of PROVEAN score to predict deleterious variation is equal to or below − 2.5, and neutral effect is above − 2.5. However, in the present research, the non-synonymous variations with PROVEAN scores above |2.5| were regarded as deleterious because we could not distinguish derived amino acid variants from the original ones at heterogenous loci. In this way we calculated the ratio of deleterious amino acid variations in heterozygous SNVs. The proportion of non-synonymous variants for each coding sequence and the proportion of nonsense SNVs (loss-of-function SNVs) to total non-synonymous SNVs were also calculated. The obtained values were compared among populations by Kruskal–Wallis test (one-way ANOVA), and as a post hoc test, multiple comparisons were conducted under Holm’s correction.

### Supplementary Information

Supplementary Figure 1.

Supplementary Tables.

## Data Availability

Raw MIG-seq data are deposited at the DDBJ Sequencing Read Archive (DRA) with accession numbers DRA015916 (Submission), PRJDB15490 (BioProject), SAMD00588474-SAMD00588501 (BioSample), DRX437804-DRX437831 (Experiment) and DRR452721-DRR452748 (Run). RNA-seq read data have been deposited at the NCBI Sequence Read Archive and are publicly available under accession number PRJDB16049.
