# Supplementary figures and images for: Double migration of the endangered Tricyrtis formosana (Liliaceae) in Japan
Source: Sci Rep. 2024 Jan 10;14:957. doi: 10.1038/s41598-024-51431-x (PMC10781951; doi:10.1038/s41598-024-51431-x)

Figure S1

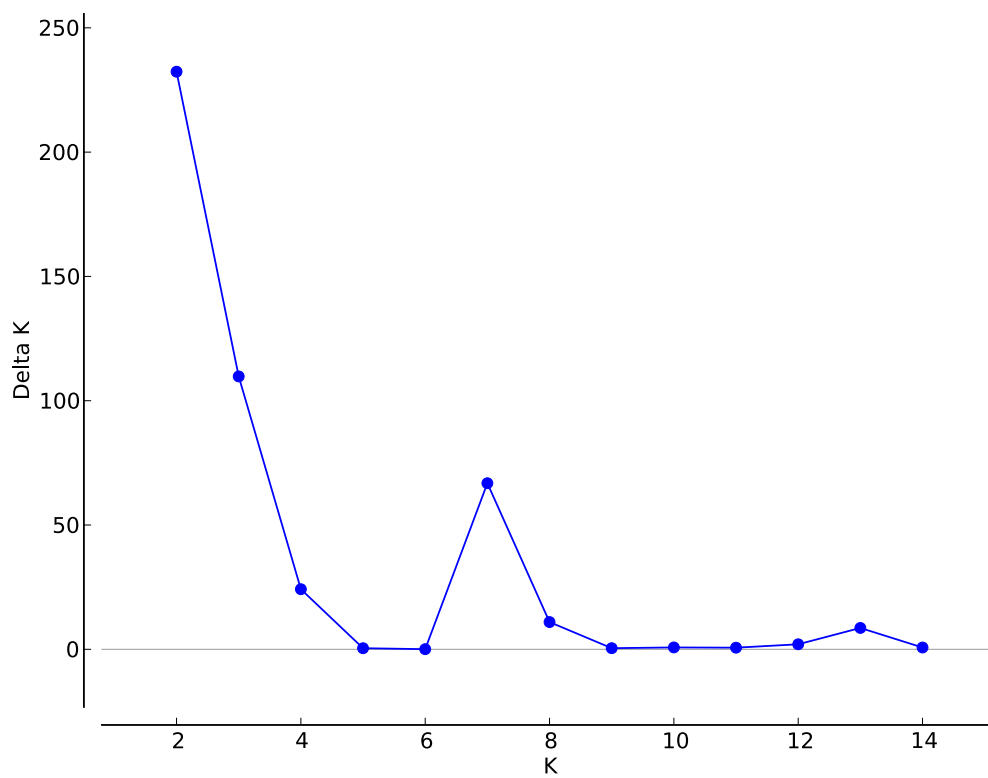

**Figure S1.** Delta K output from STRUCTURE HARVESTER.

Supplement: Supplementary file 1 — Supplementary Figure 1. [file 41598_2024_51431_MOESM1_ESM.pdf]
