# Supplementary material for: Double migration of the endangered Tricyrtis formosana (Liliaceae) in Japan
Source: Sci Rep. 2024 Jan 10;14:957. doi: 10.1038/s41598-024-51431-x (PMC10781951; doi:10.1038/s41598-024-51431-x)
Supplement: Supplementary file 2 — Supplementary Tables. [file 41598_2024_51431_MOESM2_ESM.pdf]

Table S1. Samples used for MIG-seq.

Sample ID	Species	Locality	Sampling Date
YI-1441-1	*T. formosana*	Okinawa Island (A)	Dec. 3, 2020
YI-1441-2	*T. formosana*	Okinawa Island (A)	Dec. 3, 2020
YI-1441-3	*T. formosana*	Okinawa Island (A)	Dec. 3, 2020
YI-1441-4	*T. formosana*	Okinawa Island (A)	Dec. 3, 2020
YI-1441-6B	*T. formosana*	Okinawa Island (A)	Dec. 3, 2020
YI-1441-8A	*T. formosana*	Okinawa Island (A)	Dec. 3, 2020
YI-1110-1	*T. formosana*	Iriomote Island (B)	Feb. 10, 2019
YI-0753-1	*T. formosana*	Iriomote Island (B)	Mar. 21, 2018
YI-1099-1	*T. formosana*	Iriomote Island (B)	Feb. 10, 2019
YI-1132-6	*T. formosana*	Pingxi, New Taipei (C)	Jun. 6, 2019
YI-1133-1	*T. formosana*	Pingxi, New Taipei (C)	Jun. 6, 2019
YI-1134-1	*T. formosana*	Pingxi, New Taipei (C)	Jun. 6, 2019
YI-1127	*T. formosana*	Wulai, New Taipei (D)	Jun. 6, 2019
YI-1128-6	*T. formosana*	Wulai, New Taipei (D)	Jun. 6, 2019
YI-0814-7	*T. formosana*	Datong, Yilan County (E)	Jul. 28, 2019
YI-0814-14	*T. formosana*	Datong, Yilan County (E)	Jul. 28, 2019
YI-0785-1	*T. formosana*	Sioulin, Hualien (F)	Jun. 5, 2018
YI-0785-6	*T. formosana*	Sioulin, Hualien (F)	Jun. 5, 2018
YI-0785-7	*T. formosana*	Sioulin, Hualien (F)	Jun. 5, 2018
YI-0812-17	*T. formosana*	Lugu, Nantou (G)	Jul. 26, 2018
YI-0812-27	*T. formosana*	Lugu, Nantou (G)	Jul. 26, 2018
YI-0812-28	*T. formosana*	Lugu, Nantou (G)	Jul. 26, 2018
YI-0812-15	*T. formosana*	Lugu, Nantou (G)	Jul. 26, 2018
YI-0813-9	*T. formosana*	Ren-ai, Nantou (H)	Jul. 28, 2018
YI-1207-1	*T. formosana*	Orchid Island (I)	Jul 28. 2019
YI-1152	*T. ravenii*	Alishan, Taiwan	Jun. 9, 2019

YI-0815	*T. lasiocarpa*	Lugu, Nantou y (G)	Jun. 31, 2018
YI-1111-1	Cultivar		May. 6, 2019

Table S2. Leaf samples used for measurement of leaf chlorophyll content (SPAD), cross-sectional leaf length and PAM fluorometry. NSC; Number of measured leaves (SPAD and cross-sectional leaf length), NEP; Number of measured leaves (ETR by PAM fluorometry).

Sample ID	Locality	Sampling Date	NSC	NEP
YI-1441-1	Okinawa Island (A)	Dec. 3, 2020	12	3
YI-1441-3	Okinawa Island (A)	Dec. 3, 2020	7	3
YI-1441-4	Okinawa Island (A)	Dec. 3, 2020	5	3
YI-1110-2	Iriomote Island (B)	Feb. 10, 2019	3	3
YI-1110-3	Iriomote Island (B)	Mar. 21, 2018	18	10
YI-1133-1	Pingxi, New Taipei (C)	Jun. 6, 2019	13	3
YI-1133-2	Pingxi, New Taipei (C)	Jun. 6, 2019	13	3
YI-1127-1	Wulai, New Taipei (D)	Jun. 6, 2019	8	3
YI-0814-1	Datong, Yilan County (E)	Jul. 28, 2019	12	3
YI-0814-2	Datong, Yilan County (E)	Jul. 28, 2019	19	3
YI-1146	Sioulin, Hualien (F)	Jun. 11, 2019	12	3
YI-0785-15	Sioulin, Hualien (F)	Jun. 5, 2018	8	2
YI-0785-16	Sioulin, Hualien (F)	Jun. 5, 2018	6	3
YI-0812-2	Lugu, Nantou (G)	Jul. 26, 2018	11	3
YI-0812-3	Lugu, Nantou (G)	Jul. 26, 2018	14	3
YI-0813-1	Ren-ai, Nantou (H)	Jul. 28, 2018	11	3

Table S3. Samples used for RNA-seq.

Sample ID	Locality	Sampling Date
YI-1441-2	Okinawa Island (A)	Dec. 3, 2020
YI-1441-4	Okinawa Island (A)	Dec. 3, 2020
YI-1441-6B	Okinawa Island (A)	Dec. 3, 2020
YI-1441-8A	Okinawa Island (A)	Dec. 3, 2020
YI-1110-1	Iriomote Island (B)	Feb. 10, 2019
YI-1110-2	Iriomote Island (B)	Feb. 10, 2019
YI-1110-3	Iriomote Island (B)	Mar. 21, 2018
YI-1099-1	Iriomote Island (B)	Feb. 10, 2019
YI-1216-2/3	Iriomote Island (B)	Oct. 30, 2019
YI-1133-1	Pingxi, New Taipei (C)	Jun. 6, 2019
YI-1127-1	Wulai, New Taipei (D)	Jun. 6, 2019
YI-0814-1	Datong, Yilan County (E)	Jul. 28, 2019
YI-0814-2	Datong, Yilan County (E)	Jul. 28, 2019
YI-1146	Sioulin, Hualien (F)	Jun. 11, 2019
YI-0785-15	Sioulin, Hualien (F)	Jun. 5, 2018
YI-0785-16	Sioulin, Hualien (F)	Jun. 5, 2018
YI-0812-2	Lugu, Nantou (G)	Jul. 26, 2018
YI-0812-3	Lugu, Nantou (G)	Jul. 26, 2018
YI-0813-1	Ren-ai, Nantou (H)	Jul. 28, 2018
